# The problem with composite indicators

**DOI:** 10.1136/bmjqs-2018-007798

**Published:** 2018-08-12

**Authors:** Matthew Barclay, Mary Dixon-Woods, Georgios Lyratzopoulos

**Affiliations:** 1 THIS Institute (The Healthcare Improvement Studies Institute), University of Cambridge, Cambridge, UK; 2 ECHO (Epidemiology of Cancer Healthcare and Outcomes) Group, Department of Behavioural Science and Health, University College London, London, UK

**Keywords:** quality measurement, pay for performance, report cards, health services research

## Abstract

‘The Problem with…’ series covers controversial topics related to efforts to improve healthcare quality, including widely recommended but deceptively difficult strategies for improvement and pervasive problems that seem to resist solution.

## Introduction

Increasing emphasis by policy-makers on patient choice, public accountability and quality assurance has stimulated interest in the measurement of healthcare quality and safety. A popular approach involves use of composite indicators that combine information on individual measures of care quality into single scores.^1–12^ Intended to simplify complex information, composite indicators are now widely used, for example in public reporting and in pay-for-performance schemes.^13^ Despite their ubiquity,^13 14^ they are often both problematic and controversial, for example when they are used as the basis of hospital league tables or ‘star ratings’, such as those produced by the US Centers for Medicare and Medicaid Services Hospital Compare Overall Hospital Quality Ratings (hereafter, CMS Star Ratings).^1^ In this article, we outline six common problems associated with composite indicators that seek to summarise hospital quality or safety (table 1). We use examples from different health systems and suggest possible mitigation strategies.

**Table 1:** Common issues with selected composite indicators of care quality

		CMS Overall Hospital Star Rating	AHRQ PSI90	Leapfrog Composite Patient Safety Score	MyNHS Overall Stroke Care Rating	NHS England Overall Patient Experience Score
Transparency	Are all important m ethodological details easily accessible in a public document?	Yes, but spread across several documents and web pages	Yes, but spread across several documents	Yes	No, searches of MyNHS and SSNAP websites did not find a comprehensive methods	Yes
Selection of individual measures	Are the measures used equally applicable across all rated hospitals?	No, some hospitals do not report all measures	Yes	Yes	Yes	Yes
Underlying measures and data	Is missing measure information handled in a way that can introduce bias?	Yes, pairwise deletion is used	Yes, effectively using mean imputation	Yes, pairwise deletion is used where proxy measures are not available	Yes, pairwise deletion is used	No missing measure information
	Are component measures adequately adjusted for case-mix?	Some but not all measures	Yes	Yes	Not discussed in identified methods	Yes
Use of banding onto consistent scales	Are measures standardised using banding?	No	No	No	Yes	No
Choice of weights	Is there an apparent justification for the weights used?	Yes, but reason for the precise weights used is unclear	Yes	Yes	No	No
	Is any sensitivity analysis of the choice of weights reported?	No	Yes	No	No	No
Uncertainty	Is the uncertainty in the final composite rating presented?	Not in the star rating	Yes	No	No	Yes

### Lack of transparency

Composite indicators typically seek to reduce distinct quality measures into a single summary indicator. The methods underlying such simplifications should be clear and transparent. Too often, however, composite indicators are presented with limited or no information about the derivation and interpretation of constituent measures. The technical information required to understand how composite indicators were designed is sometimes not published^5^ or is not reported alongside the actual composite indicator.^15 16^ Some measures are used without clear conceptual justification: one US scheme uses operating profit margin as a measure of quality, for example, yet why this should reasonably be seen as an indicator of (clinical) quality is not clear.^11^

Additionally, the processes by which decisions are made about what gets measured are not always clear or accountable. Clarity is needed about the role of different stakeholders in selecting measures for inclusion in composite measures, including the respective contributions of members of the public, clinicians and payers and policy-makers. This is all the more important when composite indicators are deployed as drivers of performance improvement or linked to pay-for-performance criteria.^17^

### What goes into baskets of measures matters

A key assumption underlying the use of composite indicators is that the constituent parts together give a fair summary of the whole.^17^ But composite indicators purporting to provide a broad overview of organisational quality may be dominated by a few clinical areas or by surveillance measures that are unsuitable for measuring quality. These problems may arise because of pragmatic decisions to rely on data that is readily to hand (a form of ‘availability bias’) (table 1). For example, more than one in five (15/57) of the individual underlying measures for CMS Star Ratings relate to care for cardiovascular disease, including half (8/16) of the highly weighted mortality and readmission measures.^18^ When indicators are dominated in this way by measures of specific clinical fields, they may incentivise hospitals to focus on measured disease areas at the expense of those not directly measured.^17 19 20^

Composite indicators aiming to provide broad overviews of hospital quality can also be affected by structurally absent information, such as inclusion of cardiac surgery performance measures for hospitals not providing cardiac surgery. This is not a missing data issue, rather one of irrelevance: certain performance measures are simply not applicable to particular organisations. In the CMS Star Ratings, the same methods and measures are used to produce ratings for all hospitals publicly reporting quality information on Hospital Compare,^1^ including specialty hospitals. Yet such hospitals report fewer measures than general hospitals and are substantially more likely to be classed as high-performing than the average hospital, with 87% of them receiving 4 or 5 stars in 2015 compared with 28% of all hospitals.^21^ It is plausible that the relevant subset of general quality measures do not appropriately reflect the quality of care provided by specialist hospitals.

### Threats arising from issues with underlying measures and data

Composite indicators, by their nature, obscure details about the underlying measures, yet problems in the latter can render the composite meaningless. At minimum, the underlying measures must represent valid measures of quality. To achieve this, they need to be adequately and appropriately adjusted for case-mix in order to avoid bias in the overall composite. But not all composite indicators meet these basic standards. Thus, for example, lack of adjustment for sociodemographic factors in readmission measures included the CMS Star Ratings means that hospitals serving more disadvantaged communities may receive lower ratings for reasons that are outside the hospital’s control.^22^

Problems also occur when composite indicators rely on quality measures that are not available for all hospitals. Fair comparisons rely on understanding why patient-level data are missing in order to decide whether to use a measure and, if so, how to make appropriate adjustments to reduce bias. But rates of missing data vary substantially between organisations, which may have a major impact on composite indicators.^23^ Surveillance bias, whereby organisations vary in efforts expended on collecting indicator data, may result in hospitals with the same underlying performance appearing different.^24 25^ Sometimes disclosure rules play a part in these variations. For example, some public reporting schemes purposefully suppress measures when they are based on a small number of patients or when there are data quality concerns.^26^ In other circumstances, data are simply not collected or available. The Leapfrog Hospital Safety Grade, a composite indicator of patient safety, for example, uses information from a voluntary survey of hospitals, but underlying measures are not available for hospitals that do not complete it.^27^

In practice, schemes often use *ad hoc* methods to handle missing measures, with several simply calculating ratings as the weighted average of non-missing measures.^1 10^ The CMS Star Ratings take this approach when producing overall summary scores, apparently favouring hospitals that do not provide or do not collect relevant data: hospitals that report a greater number of measured domains have systematically worse performance.^21^ It is unclear whether these differences in CMS Star Ratings reflect genuine differences or bias due to improper handling of missing variables, or improper comparisons of hospitals providing different services as discussed above under the rubric of baskets of measures.

### Banding to get measures onto consistent scales

Many composite indicator schemes apply threshold-based classification rules to standardise disparate individual measures to a consistent scale. Measures that are naturally continuous are mapped to categorical bands before being combined into the overall composite.^2 7 15^ For example, in the MyNHS Overall Stroke Care Rating, the individual measures are all mapped to 0 to 100 scales. Here, the continuous measure ‘median time between clock start and thrombolysis’ is mapped to a score of 100 if <30 min, a score of 90 if between 30 and 40 min and so on.^15^ This approach violates the general statistical principle that such categorisation reduces statistical power and potentially hides important differences.^28^ Banding distorts apparent organisational performance: hospitals with median time to thrombolysis of 29:59 would be treated as having meaningfully different performance to those with median time 30:01. These differences are unlikely to reflect reality. The thresholds used to band performance are typically arbitrary, but the particular choice of threshold can have a serious impact on estimates of organisational performance.^14 29^

The use of cliff-edge decision rules is especially unfortunate given that other ways to standardise measures without the same limitations are readily available,^8 30^ including simply applying linear interpolation between cutpoints, for example:

- Median 30 min or less receives a score of 100.
- Median 40 min exactly receives a score of 90.
- Median 37 min receives a score of $100-\left(100-90\right)\times \frac{37-30}{40-30}=93$.

### Choosing appropriate weights to combine measures

The weighting assigned to individual measures contributing to composites is another problem area. As few hospitals perform equally well in all areas, performance can be artificially improved by giving higher weight to individual measures where a hospital performs better than average and vice versa. The choice of weights given to individual measures is thus a key determinant of performance on the overall composite, and different weights might allow almost any rank to be achieved.^31 32^ Therefore, transparency is needed about the importance attached to each measure in terms of the aim of the indicator, with supporting evidence. However, many schemes do not provide explicit justification for the weights used to create the composite (table 1). Not assigning any weights is also fraught with problems. The NHS England Overall Patient Experience Scores scheme does not allocate different weights to survey questions because ‘there was no robust, objective evidence base on which to generate a weighting’.^6^ But that criticism is also applicable to the decision to adopt equal weights.^33^ Similarly, the composite patient safety indicator AHRQ PSI90, since revised,^34 35^ originally gave greater weight to more common safety incidents,^10^ ignoring differences in the degree of potential harm to patients. The original specification gave a 18-fold greater weight to the incidence of pressure ulcers compared with postoperative hip fracture.^34^

Patient-level composite indicators have various advantages and drawbacks, well summarised in the clinical trial literature.^36^ However, appropriate prioritisation of individual measures at patient-level is vital. Consider the so-called ‘textbook outcome’ approach proposed by Kolfschoten and colleagues following colon cancer resection.^37^ A ‘textbook outcome’ is one where a patient has the ideal outcomes after resection, so patients score 0 if they have any negative outcome (extended stay in hospital, surgical complication, readmission, death and so forth) and 1 otherwise. Giving the same importance to an extended stay in hospital and to death is not justified. Instead, the approach should reflect the relative importance of each outcome, for example by ranking the different possible outcomes in terms of degree of potential clinical harm or patient preferences.^38^

### Failure to present uncertainty

Composite indicators are not immune to chance variation: tiny differences in individual measures can translate into differences in the final rating, but will often be due to chance.^39^ Simulations show that around 30% of US hospitals might be expected to change CMS Star Rating from year-to-year due to chance alone.^1^ Yet many composite indicators are presented without appropriate measures of uncertainty (table 1), in defiance of expert recommendation and established practice for individual performance measures.^30 40–42^ Of course, confidence intervals spanning multiple performance categories might lead users to view an indicator as meaningless: when comparing performance between two hospitals, it is easier to say one is three-star and the other four-star, rather than say that one is ‘between two and four stars’ and the other is ‘between three and five stars’. However, when there is a lot of uncertainty about hospital performance, hospitals should not be penalised or rewarded for performance that may simply reflect the play of chance—making it especially important that reporting conventions are well-founded.

## Possible solutions

Though the clamour about flawed composite measures and their role in comparing organisations is growing louder,^13 17 22 23 43–45^ they continue to be widely deployed. Rather than repeating existing principled frameworks for developing composites,^33 46^ we highlight a few sensible approaches (table 2) and discuss areas for further research.

**Table 2:** Requirements, steps forward and remaining challenges for robust and useful composite indicators

Requirement	Steps forward	Remaining challenges
Transparency *The principles and theory underlying the composite indicator must be clear*	Being clear about who is involved in making decisions in developing the composite indicator.	Many stakeholders may be involved. The design may evolve in unexpected ways over time.
	Fully describing the decision-making process, reporting the reasons and justifications for the decisions made.	
Purpose-led design *The composite indicator must plausibly measure what it sets out to measure*	Selecting individual measures to cover the full range of services intended to be measured by the composite.	Identifying appropriate individual measures. Appropriate measures may not exist for all areas included in the composite.
	Choosing weights that reflect the relative importance of the different quality measures.	Balancing the weighting system against competing priorities.
Technical reproducibility *The composite indicator must be reproducible using the raw data and the published methodology*	Providing clear and comprehensive technical documentation.	
	Reporting full definitions of the individual underlying measures and how they are combined.	Individual measures may only be available from sources that do not fully document the details, but these measures should not be used in the composite.
	Publishing the code used in data processing and statistical analysis.	
Statistical fitness *Individual measures must be adequately adjusted for case-mix, have acceptable statistical reliability and be appropriately standardised to consistent scales*	Performing appropriate statistical case-mix adjustment.	Accurate patient-level data may not exist for important case-mix factors. Adequate statistical case-mix adjustment may not be possible. Interpretable results may require further processing.
	Using reporting periods long enough to give acceptable reliability.	Longer reporting periods may be necessary to increase reliability, but impedes use in driving quality improvement.
	Standardising measures to consistent scales in a principled way that preserves the useful information in the underlying measures.	Understanding what good and bad performance in the real world looks like on each measure.

We propose that methodological transparency is key to addressing many current problems with composite measures. The aims and limitations of composite indicators should be presented alongside ratings to aid understanding of where scores and ratings come from, what they mean and what limits their usefulness or interpretability. Methodological information should be readily available and clearly linked to the indicator. Clear explanation is needed of the logic underlying the development of each composite indicator, including the choice of measures, any compromises between different goals, whose views have been taken into account in producing the indicator and how. Many composite indicators would be improved by reflecting the aims and preferences of the relevant stakeholders in the choice and weighting of individual measures using a clear process and explicit theory-of-change.^47–50^

An important element of transparency is that composite indicators are presented with accompanying displays of statistical uncertainty.^30^ Uncertainty in composite indicators arises both from statistical noise and from the way individual measures are chosen, standardised and aggregated. Sensitivity analyses should investigate whether reasonable alternative methods would substantially alter organisational rankings,^40^ and the results of these analyses should be reported.^31^ This may require addressing the current lack of scientific consensus about how best to represent uncertainty for star-ratings and other categorical performance classifications. Interval estimates, such as confidence intervals, are the typical way of representing uncertainty and can certainly be calculated for ranks and scores on composite indicators.^31^ They may be less useful for indicators presented as star-ratings; it may be better to discuss the probability that a rating is correct, or too high or low, drawing on Bayesian approaches to ranking hospital performance on individual measures.^51^ One alternative is to build a formal decision model based on the harm caused by misclassifying a hospital as better or worse than it is,^52 53^ but in practice this may raise further problems relating to how harms are judged.

Composite indicators should be designed in accordance with good statistical practice. Underlying measures should, at minimum, be appropriately adjusted for case-mix, assessed for possible sources of bias and meet basic standards of interunit reliability.^40 54 55^ The reasons for missing data should be explored, and principled approaches should be adopted to address missing data. Entirely missing measures (eg, a hospital has no thrombolysis time information at all) may sometimes be handled using statistical approaches to identify common factors between measures based on the observed hospital-level correlations.^56–58^ Missing data in individual measures (eg, 30% of patients at a given hospital have missing thrombolysis time) may sometimes be handled using multiple imputation to predict what missing values should have been based on the available information.^59 60^ The likely best solution is to refine inclusion criteria and improve data collection so that the proportion of missing data becomes negligible.

Individual measures must be on the same scale before they can meaningfully be combined into an overall composite. This often requires measures to be standardised. There are many methods of standardising collections of measures, and here methodological choices need guiding by an understanding of clinical best practice and the meaning of differences in performance on the individual scales. Often, it may simply be that ‘higher is better’, and so default approaches may be optimal. One default option is to standardise against the observed standard deviation (‘Z-scoring’),^30^ with the standardised measure describing how far a given hospital’s performance is from the average hospital, relative to variation across all hospitals. Another option is to standardise against the possible range of measure scores, so the standardised value describes how close a hospital is to achieving the theoretical maximum performance. But it is often possible to modify these defaults to produce a more meaningful composite, perhaps by measuring performance relative to targets or by incorporating information about the importance of achieving particular levels. In particular, it may be possible for some measures to identify clear thresholds for acceptable, good and excellent performance on a measure, as for example for some component measures of the MyNHS Overall Stroke Care Rating.^15^ Interpolation between thresholds allows standardisation to a meaningful scale without the use of cliff-edge decision rules.

Modern data visualisation techniques may help make composite indicators more informative and useful in healthcare, perhaps building on emerging examples of composite measures and rankings outside of healthcare where the user can interactively specify measure weights on a web page and immediately see the impact on results.^61^ This may allow users to make composites that reflect their own priorities and to explore uncertainty due to the way measures are aggregated. But poorly designed visualisation may mislead users or require more effort to understand than less attractive options. Research focused on the design designs and benefits and harms of different data visualisation strategies for performance measurement is vital.

## Conclusion

Composite indicators promise a simple, interpretable overview of complex sets of healthcare quality information. But that may be an empty promise unless the problems we describe here are addressed. Implementing improvements to the design and reporting of composite indicators and other performance measures requires concerted effort to promote higher levels of scrutiny of decisions about individual measures of quality, their related technical specification and standards. Health systems should have clearly defined processes for ensuring new performance measures are relevant, useful and scientifically sound. These should incorporate periodic reviews of all measures, so that those found to be no longer relevant or useful are either withdrawn or appropriately revised. Reporting guidelines support clear and transparent reporting of the design of these indicators are likely to be a useful next step.

- Composite indicators aim to provide simple summary information about quality and safety of care.
- Many current composite indicators suffer from conceptual and statistical flaws that greatly limit their usefulness, though most such flaws can be addressed.
- Much greater transparency is needed about the goals that different composite indicators intend to achieve.
- Guidelines about the development, design and reporting of composite indicators are likely to be of benefit.
